# Alcohol Consumption and Associated Factors Among Elderly People in Central Brazil

**DOI:** 10.1093/geroni/igaf122.3538

**Published:** 2025-12-31

**Authors:** Larissa Magalhaes, Milara Barp, Bruno Vinícius Diniz e Silva, Sheila Teles, Erika Aparecida Silveira, Winny Eveny Moura, Pao-Feng Tsai, ´Valeria Pagotto

**Affiliations:** Auburn University, Auburn, Alabama, United States; Federal University of Goias, Goiania, Goias, Brazil; Federal University of Goias, Goiania, Goias, Brazil; Federal University of Goias, Goiania, Goias, Brazil; Federal University of Goias, Goiania, Goias, Brazil; Federal University of Goias, Goiania, Goias, Brazil; Auburn University, Auburn, Alabama, United States; Federal University of Goias, Goiania, Goias, Brazil

## Abstract

**Background:**

Alcohol is a legal substance widely consumed across different social strata and age groups. Among older adults, alcohol use poses growing challenges for health management due to age-related physiological vulnerability. This study aimed to estimate alcohol consumption patterns and identify associated risk factors in older adults.

**Methods:**

A survey was conducted in Goiânia, Central-West Brazil, from July 2018 to March 2019, using the Alcohol Use Disorders Identification Test (AUDIT). Participants were part of the second wave of a cohort study. A total of 221 older adults were interviewed and included in the analysis.

**Results:**

Among the participants, 14.9% reported alcohol use in the past year. Most showed low-risk consumption patterns (97.3%), and 1.8% reported binge drinking. Gender (adjusted odds ratio [aOR] = 2.57), religion (aOR = 0.45), and education level (aOR = 0.18) were significantly associated with alcohol use.

**Conclusions:**

Male gender was associated with greater alcohol use, while higher education and religious affiliation appeared protective. Although the prevalence of alcohol consumption was low in older adult population, the findings highlight the need for targeted actions in older men. These results contribute to understanding the health behaviours of older adults and emphasize the importance of tailored health promotion strategies considering the heterogeneity of aging.

